# Anti-*Candida* properties of asaronaldehyde of *Acorus gramineus* rhizome and three structural isomers

**DOI:** 10.1186/1749-8546-8-18

**Published:** 2013-09-08

**Authors:** Sandeep B Rajput, Ravikumar B Shinde, Madhushree M Routh, Sankunny M Karuppayil

**Affiliations:** 1 DST-FIST and UGC-SAP Sponsored School of Life Sciences, SRTM University, Nanded 431-606, MS, India

## Abstract

**Background:**

Asaronaldehyde (2, 4, 5-trimethoxybeznaldehyde) is an active component of *Acorus gramineus* rhizome. This study aims to evaluate the anti-*Candida* efficacy of asaronaldehyde and its three structural isomers, namely, 2, 3, 4-trimethoxybenzaldehyde, 3, 4, 5-trimethoxybenzaldehyde, and 2, 4, 6- trimethoxybenzaldehyde.

**Methods:**

Susceptibility testing of test compounds was carried out using standard methodology (M27-A2) as per clinical and laboratory standards institute guidelines. Minimum fungicidal concentration (MFC) was determined as the lowest concentration of drug killing 99.9% of *Candida* cells. The effect on sterol profile was evaluated using the ergosterol quantitation method. Effects on morphogenesis, adhesion and biofilm formation in *C. albicans* were studied using germ-tube, adherence and biofilm formation assays respectively. Cytotoxicity of test compounds to human RBCs was determined by hemolysis assay.

**Results:**

2, 4, 6-Trimethoxybenzaldehyde exhibited significant anti-*Candida* activity (*P* = 0.0412). Minimum inhibitory concentration (MIC) and minimum fungicidal concentration (MFC) were established as 0.25 and 0.5 mg/mL, respectively. All of the test compounds showed significant inhibition of hyphal form transition in yeast at MIC/2 and MIC/4 values. 3, 4, 5-Trimethoxybenzaldehyde and 2, 4, 6-trimethoxybenzaldehyde inhibited adhesion and biofilms. A hemolytic assay of these compounds revealed that they were non-toxic at MIC values. Asaronaldehyde reduced sterol content.

**Conclusion:**

Asaronaldehyde and 2, 4, 6-trimethoxybenzaldehyde showed anti-*Candida* efficacy.

## Background

Drug resistant pathogens and serious side effects are major problems in antifungal chemotherapy [1]. *Candida* species are the fourth leading cause of nosocomial bloodstream infections in the United States, with treatment costs estimated to be more than two to four billion US dollars annually [2] and with attributable mortality rates estimated to be 38–49% [3]. *Candida albicans* is a ubiquitous organism in humans and causes serious disseminated infections in the immunocompromised population [4]. It colonizes and forms biofilms on host tissues and indwelling prosthetic devices [5]. Biofilms are resistant to most of the available antibiotics, except echinocandins (*e.g.*, caspofungin and micafungin) [6]. Several antifungal compounds have been developed, targeting diverse biological pathways essential for fungal growth, *e.g.*, the ergosterol synthesis pathway or its end product ergosterol [7]. Unfortunately, most of the available antifungals have side effects, such as nephrotoxicity (amphotericin B) and gastrointestinal intolerance (Fluconazole), and drug resistance has developed [8-10]. The emergence of drug resistance and serious side effects suggest the need for new and safer antifungals.

**Figure 1:**
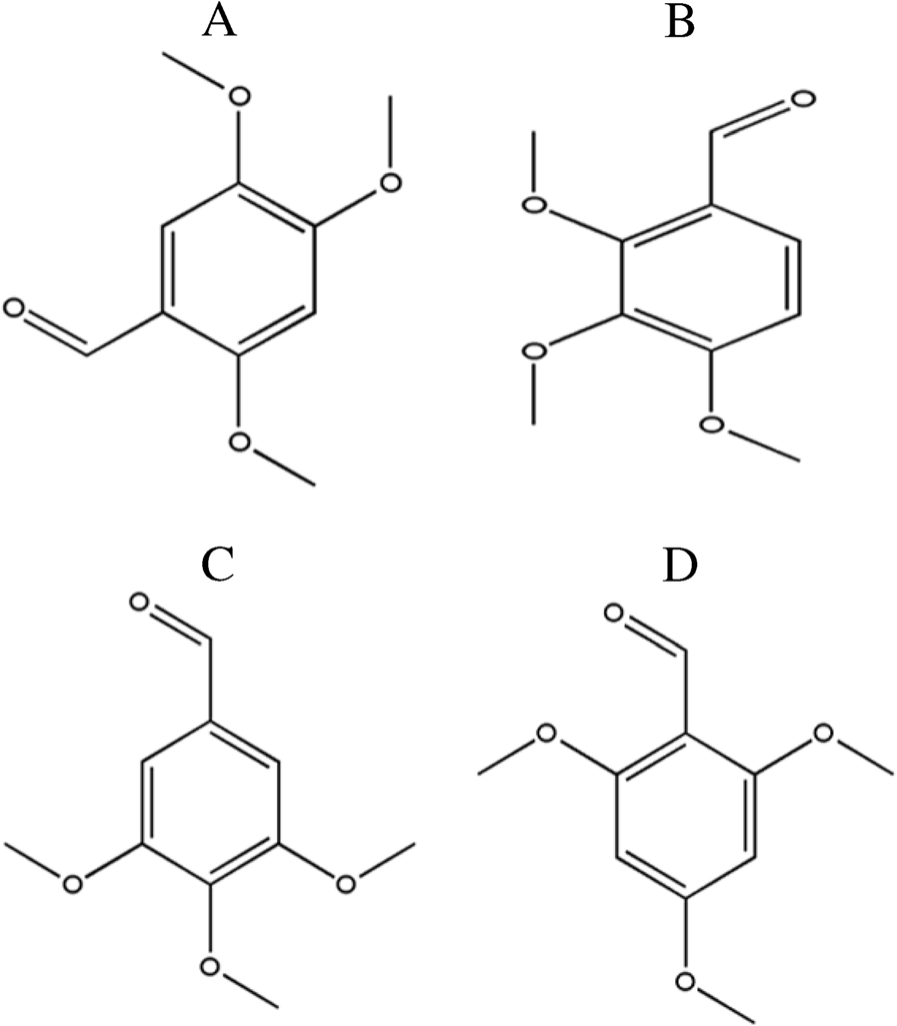
Chemical structures of A: 2,4,5-trimethoxybenzaldehyde, B: 2,3,4- trimethoxybenzaldehyde, C: 3,4,5-trimethoxybenzaldehyde and D: 2,4,6-trimethoxybenzaldehyde.

Chinese and Korean pharmacopoeias have demonstrated sedative, digestive, analgesic, diuretic and antifungal actions of extracts of *Acorus gramineus*[11-14]. Asaronaldehyde (2, 4, 5-trimethoxybeznaldehyde), an active principle of *A. gramineus* rhizomes, exhibits fungicidal activity against several phytopathogenic fungi [15]. Asaronaldehyde is a selective inhibitor of cyclooxygenase II (COX-II) [16], consisting of a benzene ring substituted with aldehyde and methoxy groups. Aromatic compounds bearing a trimethoxy group are known to exhibit good antibacterial and antifungal activities [17]. However, structurally related compounds differ in their bioactivities, and the difference in the conformation and position of the functional group(s) determines their binding affinity to target molecules and clinical efficacy [18,19]. To our knowledge, the effects of asaronaldehyde and its structural isomers, namely, 2, 3, 4-trimethoxybenzaldehyde, 3, 4, 5-trimethoxybenzaldehyde, and 2, 4, 6-trimethoxybenzaldehyde (Figure 1), on the human pathogenic yeast *C. albicans*, have not been reported. However, alpha and beta asarones are known to exhibit anti-*C. albicans* activity [20].

This study aimed to investigate the anti-*Candida* efficacy and mechanism of action of asaronaldehyde and its structural isomers.

## Methods

### Media, chemicals and culture conditions

The *C. albicans* ATCC 90028 (MTCC 3017) strain was obtained from the Institute of Microbial Technology (Chandigarh, India), and was maintained on Yeast–Peptone–Dextrose (YPD) agar slants at 4°C. RPMI-1640 medium (with L-glutamine and without sodium bicarbonate), 3-[N-morpholine] propane sulfonic acid (MOPS), Sabourauds dextrose broth (SDB), YPD media and 3-(4, 5-dimethylthiazol-2-yl)-2, 5-diphenyltetrazolium bromide (MTT) were purchased from Hi-Media Laboratories Ltd. (Mumbai, India). Triton-X 100 was purchased from Qualigens Fine Chemicals Pvt. Ltd. (India). 2, 4, 5-Trimethoxybenzaldehyde (asaronaldehyde or asaraldehyde), 2, 3, 4-trimethoxybenzaldehyde, 3, 4, 5-trimethoxybenzaldehyde, and 2, 4, 6-trimethoxybenzaldehyde were purchased from Thermo Fisher Scientific (Acros Organics), Pvt. Ltd. (India).

### Growth assay

The susceptibility study was carried out using the microbroth dilution method as per clinical and laboratory standards institute (CLSI) guidelines [21]. Various concentrations of the test compounds (0.0156 mg/mL to 8 mg/mL) were prepared in RPMI-1640 medium by double dilution in 96-well plates. Each well contained an inoculum of 1 × 10^3^ cells mL^-1^ and the final volume of RPMI-1640 medium maintained in each well was 200 μL. Wells without addition of compounds served as a control. Microplates were incubated at 35°C for 48 h and read spectrophotometrically at 620 nm using a microplate reader (Multiskan EX, Thermo Electron Corp. USA). The minimum inhibitory concentration (MIC) was considered to be the minimum concentration required to produce a 50% reduction in absorbance.

### Viability assay

Cells from the MIC and the wells above were selected to determine the minimum candidacidal concentrations of the test compounds. An aliquot of 10 μL of cell suspension from each well was spread on YPD agar plates. The plates were incubated for 24 h at 30°C and observed for the presence of colonies. Minimum fungicidal concentration (MFC) was considered as the lowest concentration required to kill 99.9% of *Candida* cells [22].

### Hemolytic activity

The hemolytic activities of the test compounds were determined using human red blood cells [23]. Human erythrocytes from healthy persons were collected in tubes containing EDTA (1–2 mg/ml) as anti-coagulant. The erythrocytes were harvested by centrifugation (Heraeus Megafuge 40, Thermo Fisher Scientific Inc., MA) for 10 min at 634 × *g* at 20°C, and washed three times in PBS. PBS was added to the pellet to yield a 10% (v/v) erythrocytes/PBS suspension. The 10% suspension was diluted 1:10 in PBS. From each suspension, 100 μL was added in triplicate to 100 μL of a different dilution series of test compounds (or fluconazole as a standard antifungal) in the same buffer in Eppendorf tubes. Total hemolysis was achieved using 1% Triton X-100. The tubes were incubated for 1 h at 37°C and centrifuged for 10 min at 634 × *g* at 20°C. From the supernatant fluid, 150 μL was transferred to a flat-bottomed microtiter plate (Tarson India Ltd., India), and the absorbance was measured at 450 nm. Percent hemolysis was calculated using following equation:

$$\begin{array}{c} \%\mathrm{Hemolysis}\\ \;=\frac{A_{450}\mathrm{of}\;\mathrm{test}\;\mathrm{compound}\;\mathrm{treated}\;\mathrm{sample}-A_{450}\mathrm{of}\;\mathrm{buffer}\;\mathrm{treated}\;\mathrm{sample}}{A_{450}\mathrm{of}\;1\;\%\;\mathrm{Triton}-X\;100\;\mathrm{treated}\;\mathrm{sample}-A_{450}\mathrm{of}\;\mathrm{buffer}\;\mathrm{treated}\;\mathrm{sample})}\\ \;\times 100\;\% \end{array}$$

### Ergosterol extraction and quantitation assay

A single colony of *C. albicans* from an overnight grown SDA plate culture was used to inoculate 50 mL of SDB containing various concentrations of different test compounds. SDB without test compound served as a control. The cultures were incubated for 16–18 h and harvested by centrifugation at 856 × *g* for 5 min. The net weight of the cell pellet was determined. Three milliliters of 25% alcoholic potassium hydroxide solution was added to the cell pellet and vortex mixed for 1 min. Cell suspensions were transferred to sterile borosilicate glass screw-cap tubes and incubated in an 85°C water bath for 1 h. Following incubation, the tubes were cooled. Sterols were extracted by addition of a mixture of 1 mL of sterile distilled water and 3 mL of *n*-heptane followed by vigorous vortex mixing for 3 min. The heptane layer was transferred to a clean borosilicate glass screw-cap tube. Prior to analysis, a 0.6 mL aliquot of sterol extract was diluted five fold in 100% ethanol and scanned between 240 and 300 nm using a spectrophotometer (UV-1800 Spectrophotometer, Shimadzu, Japan). Ergosterol content was calculated as a percentage of the wet weight of the cell [24].

### Germ tube formation assay

Inhibition of *in vitro* germ tube formation by test compounds was studied using 96-well microtiter plates based on the method of Chauhan *et al.*[25]. Cells were inoculated in RPMI-1640 medium with various concentrations of test compounds to obtain 1 × 10^6^ cells/mL. Wells without test compound were used as a control. The plates were incubated at 37°C with shaking at 200 rpm on an orbital shaker for 3 h, and cells were observed microscopically. Each time, 100 cells were counted and the numbers of yeast cells (budded or unbudded) and germ tubes formed were noted. The percentage of germ tube formation in each well compared with that in the control well was calculated.

### Adhesion assay

The effects of the four test compounds on adherence of *C. albicans* to a polystyrene surface were studied based on the method of Camacho *et al.*[26]. In brief, 1 × 10^7^ cells/mL was allowed to adhere to the polystyrene surface of 96-well plates in the presence of various concentrations ranging from 0.0156 mg/mL to 2 mg/mL of the test compounds in PBS. Wells without test compound were used as controls. The plates were incubated at 37°C for 90 min at 100 rpm on an orbital shaking incubator to allow cell attachment to the surface. After the incubation, wells were washed with PBS to remove non-adhered cells. The density of adherence in each well was analyzed using an MTT metabolic assay and the percentage of adhered cells was calculated and compared with that in control wells. MTT solution was prepared by mixing 1 mg/mL MTT salt in PBS and storing at -20°C.

### Biofilm formation assay

*C. albicans* biofilms were developed on polystyrene surfaces of 96-well plates as per standard methodologies [27]. One hundred microliters of a cell suspension (1 × 10^7^ cells/mL) in PBS was inoculated and plates were incubated at 37°C for 90 min to allow attachment of cells onto the surface. Non-adhered cells were removed by washing the wells with sterile PBS, two to three times. RPMI-1640 medium (200 μL) was added to each well and the plates were incubated at 37°C for 24 h to allow biofilm formation. RPMI-1640 medium with various concentrations of test compound was added immediately after the adhesion phase to observe any effects on the development of biofilms. After incubation, wells were washed to remove any released cells, and biofilms were observed under an inverted light microscope (Metzer, India). Biofilm growth was analyzed by MTT-metabolic assay.

### Biofilm quantitation by MTT assay

Biofilm growth was quantified by MTT metabolic assay [27]. Wells containing biofilms were washed with PBS to remove non adhered cells and incubated for 5 h in 100 μL of MTT solution in the dark. After incubation, unbound MTT was replaced with 150 μL of DMSO (100%) for 2 to 3 min. Treated samples (100 μL) were then taken into new wells. Color formation was measured at 450 nm using a microplate reader (Multiskan EX, Thermo Electron Corp. USA). Wells without test compounds were used as controls, while those without biofilms were the blanks.

### Scanning electron microscopy (SEM) of *C. albicans* biofilms

*Candida* biofilms were developed by seeding silicon discs with 2 mL of standardized cell suspension of 1 × 10^7^cells/mL into 12-well plates. The plates were incubated at 37°C at 50 rpm for 90 min. Non-adhered cells were removed by washing the discs with PBS 2–3 times. RPMI-1640 medium along with 2, 4, 6-TMB (0.5 mg/mL) and one control without 2, 4, 6-TMB was added to each well. The final volume of the assay system in each well was kept to 3 mL. The plate was incubated at 37°C for 24 h to allow biofilm formation. After incubation, discs were washed to remove any planktonic cells. For SEM, samples were fixed in 2.5% of glutaraldehyde (0.1 mol/L) in phosphate buffer (pH 7.2) for 24 h at 4°C. Samples were postfixed in a 2% aqueous solution of osmium tetraoxide for 4 h, then dehydrated in a series of graded alcohols and finally dried to a critical drying point with a Critical Point Dryer unit. The samples were mounted over stubs and gold coating was performed by an automated gold coater (Model: JOEL JFC-1600, JOEL Limited, Akishima, Tokyo 1960022, Japan) for 3 min. Photos were taken under a scanning electron microscope (Model: JOEL JSM 7600F, JOEL Limited, Akishima, Tokyo 1960022, Japan) [25].

### Statistical analysis

Values are presented as the means of triplicate observations ± standard deviation (SD). Percentage growth in the presence of test compounds and in controls was analyzed by Student’s *t* test (GraphPad prism 5.0 software, USA), and *P* < 0.05 was considered statistically significant. The concentration-dependent manner of any effect was visually determined.

## Results and discussion

**Figure 2:**
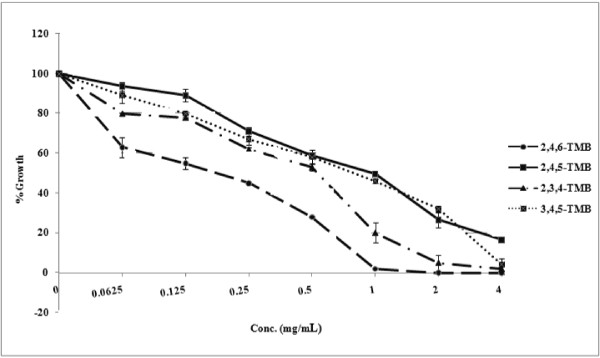
**Effects of 2,4,5-trimethoxybenzaldehyde; 2,3,4-trimethoxybenzaldehyde; 3,4,5-trimethoxybenzaldehyde and 2,4,6-trimethoxybenzaldehyde on the growth of *C. albicans *ATCC 90028.**

The compounds tested in this study, which differ only in terms of the conformation or position of methoxy groups, showed various degrees of antifungal activity. All of the compounds significantly (*P* = 0.0412 for 2, 4, 5-trimethoxybenzaldehyde, *P* = 0.0454 for 2, 4, 5-trimethoxybenzaldehyde, *P* = 0.0490 for 2, 3, 4-trimethoxybenzaldehyde, *P* = 0.0475 for 3, 4, 5-trimethoxybenzaldehyde) inhibited planktonic growth of *C. albicans* in a concentration-dependent manner. 2, 4, 6-trimethoxybenzaldehyde inhibited the growth and viability of *C. albicans* at 0.25 and 1 mg/mL, respectively, while 2, 4, 5-trimethoxybenzaldehyde, 2, 3, 4-trimethoxybenzaldehyde and 3, 4, 5-trimethoxybenzaldehyde showed MIC at 1 mg/mL (Figure 2). The MFC of 2, 3, 4-trimethoxybenzaldehyde was 2 mg/mL, whereas the MFCs for 2, 4, 5-trimethoxybenzaldehyde and 3, 4, 5-trimethoxybenzaldehyde were 8 and 4 mg/mL, respectively.

**Figure 3:**
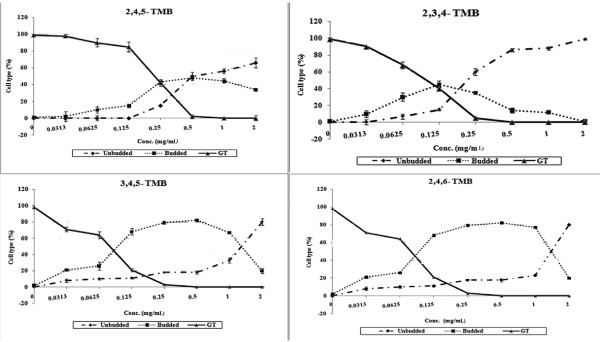
**Effects of 2,4,5-trimethoxybenzaldehyde; 2,3,4-trimethoxybenzaldehyde; 3,4,5-trimethoxybenzaldehyde and 2,4,6-trimethoxybenzaldehyde on RPMI-1640-induced germ tube formation in *C. albicans *ATCC 90028.**

**Figure 4:**
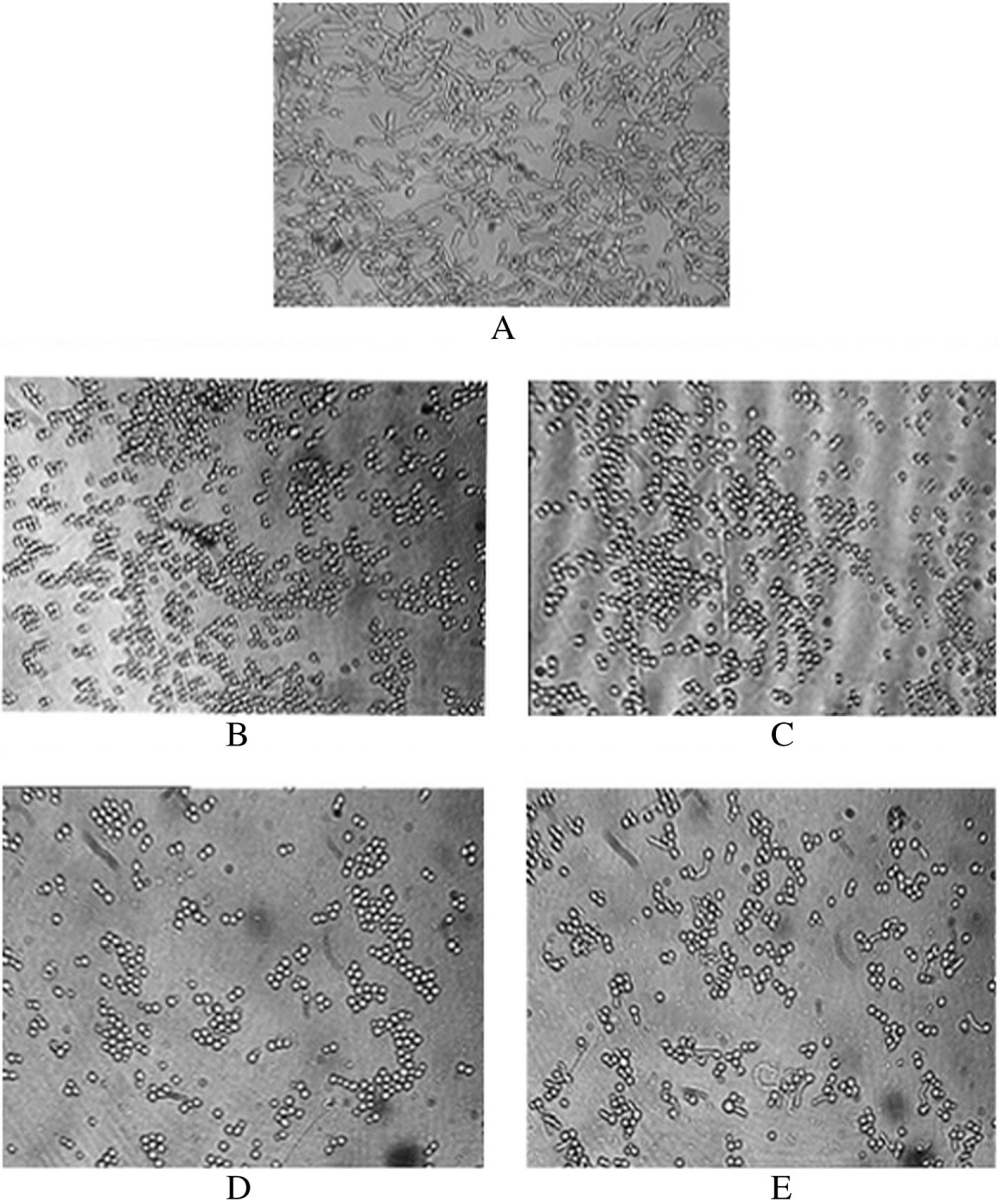
**Germ tube formation of *C. albicans *ATCC 90028 in the absence of the test compound (A) and in the presence of an MIC/2 concentration of 2,4,5-trimethoxybenzaldehyde (B), 2,3,4-trimethoxybenzaldehyde (C), 3,4,5-trimethoxybenzaldehyde and (D) 2,4,6-trimethoxybenzaldehyde (E).**

Complete inhibition of *Candida* growth by the four compounds was observed at sub-inhibitory concentrations (Figure 3) (*P* = 0.0402 for 2, 4, 5-trimethoxybenzaldehyde, *P* = 0.0410 for 2, 4, 5- trimethoxybenzaldehyde, *P* = 0.0480 for 2, 3, 4-trimethoxybenzaldehyde, and *P* = 0.0497 for 3, 4, 5-trimethoxybenzaldehyde). All four compounds inhibited 80–100% of the yeast to hyphal transition at sub-inhibitory concentrations (*P* = 0.0353 for 2, 4, 5-trimethoxybenzaldehyde, *P* = 0.0301 for 2, 4, 5-trimethoxybenzaldehyde, *P* = 0.0390 for 2, 3, 4-trimethoxybenzaldehyde, and *P* = 0.0405 for 3, 4, 5-trimethoxybenzaldehyde). In addition to germ tube inhibition, inhibition of budding was also recorded (Figures 3 and 4). A greater than 50% of reduction in morphogenesis was observed at 0.125 mg/mL for 2, 3, 4-trimethoxybenzaldehyde, 3, 4, 5-trimethoxybenzaldehyde and 2, 4, 6-trimethoxybenzaldehyde, while similar results were observed at 0.25 mg/mL for 2, 4, 5-trimethoxybenzaldehyde. Inhibiting virulence factors, *e.g.*, dimorphism, without killing the pathogen, might avoid natural selection and thereby prevent the emergence of a drug-resistant population.

**Figure 5:**
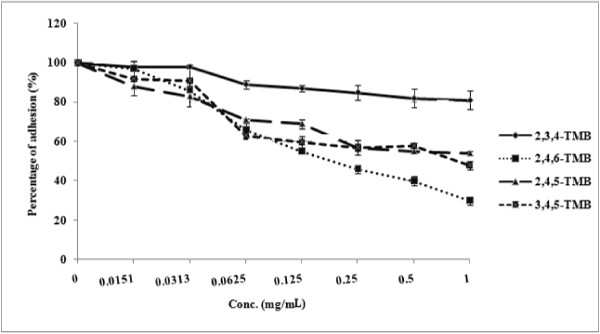
**Effects of 2,4,5-trimethoxybenzaldehyde; 2,3,4-trimethoxybenzaldehyde; 3,4,5-trimethoxybenzaldehyde and 2,4,6-trimethoxybenzaldehyde on adhesion in *C. albicans *ATCC 90028.**

**Figure 6:**
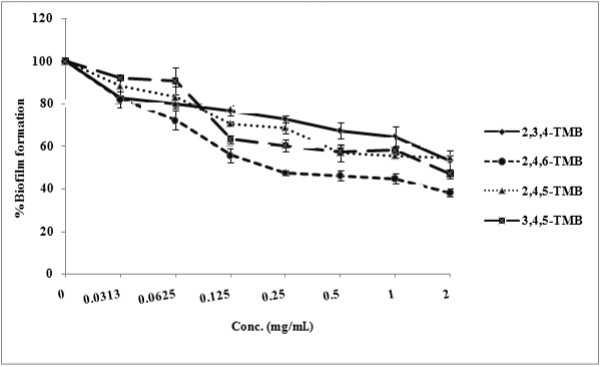
**Effects of 2,4,5-trimethoxybenzaldehyde; 2,3,4-trimethoxybenzaldehyde; 3,4,5-trimethoxybenzaldehyde and 2,4,6-trimethoxybenzaldehyde on biofilm formation in *C. albicans *ATCC 90028.**

Adhesion is an early stage of biofilm development which is important for *Candida* colonization and infection [5]. The compounds 3, 4, 5-trimethoxybenzaldehyde and 2, 4, 6-trimethoxybenzaldehyde significantly inhibited biofilm development in *C. albicans* (*P* = 0.050 for 3, 4, 5-trimethoxybenzaldehyde, and *P* = 0.0495 for 2, 4, 6-trimethoxybenzaldehyde) (Figures 5 and 6)*.* These compounds prevented more than 50% of adhesion at their MIC values (*P* = 0.0481 for 3, 4, 5-trimethoxybenzaldehyde, and *P* = 0.0395 for 2, 4, 6-trimethoxybenzaldehyde). The compound 2, 4, 5-trimethoxybenzaldehyde showed a 46% reduction in adhesion at 1 mg/mL (*P* = 0.05). However, 2, 3, 4-trimethoxybenzaldehyde did not have a considerable effect on the adhesion of *C. albicans* yeast-phase cells (*P* = 0.0481); only 19% inhibition of adhesion was observed at MIC (Figure 5).

**Figure 7:**
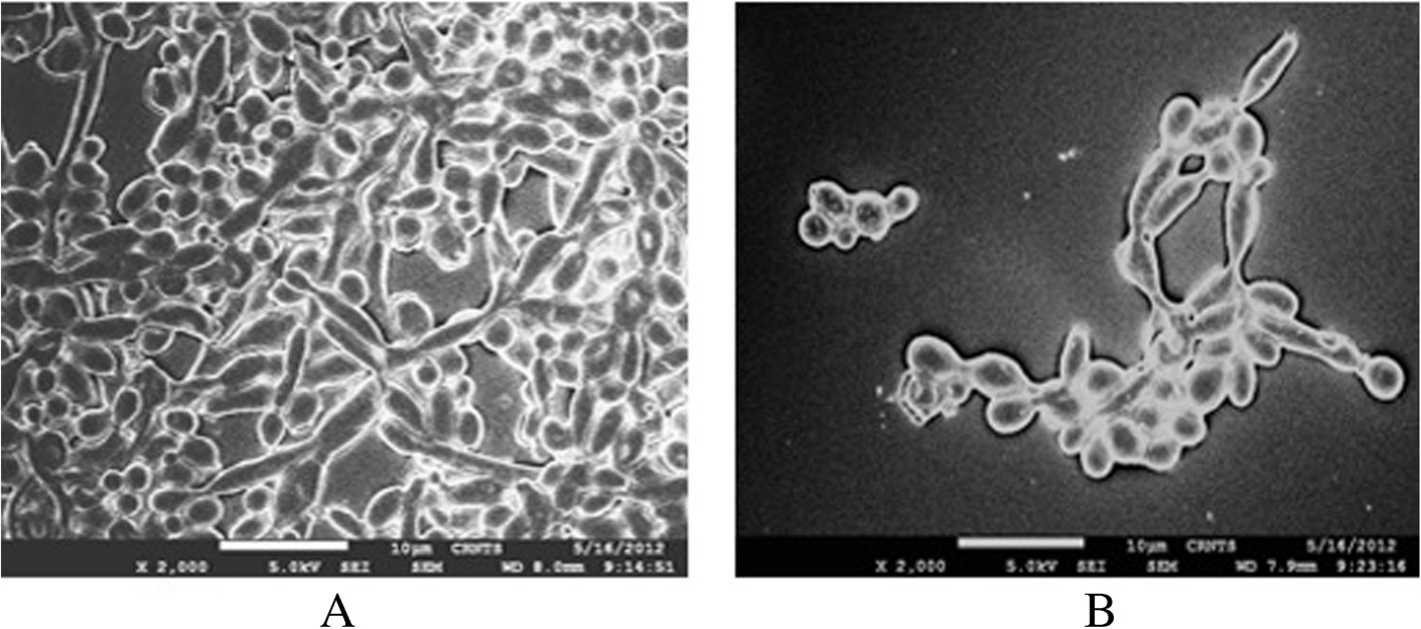
**Scanning electron micrograph (at magnification × 2000) of a *Candida albicans *biofilm in the absence (A) and presence (B) of 2 × MIC of 2, 4, 6-trimethoxybenzaldehyde.**

Biofilms are resistant to antifungal antibiotics, including the most commonly prescribed drug, fluconazole [5,28]. All of the compounds tested significantly inhibited biofilm formation, even at their MIC values. The MIC for prevention of biofilm formation for 3, 4, 5-trimethoxybenzaldehyde and 2, 4, 6-trimethoxybenzaldehyde was achieved at the MIC for inhibition of growth (*P* = 0.05 for 3, 4, 5-trimethoxybenzaldehyde, and *P* = 0.0495 for 2, 4, 6-trimethoxybenzaldehyde). Addition of 1 mg/mL 2, 4, 5-trimethoxybenzaldehyde and 2, 3, 4-trimethoxybenzaldehyde immediately after the adhesion phase caused 45% and 35% inhibition of biofilm formation, respectively (*P *= 0.0377 for 2, 4, 5-trimethoxybenzaldehyde, and *P* = 0.0475 for 2, 3, 4-trimethoxybenzaldehyde) (Figure 6). The ultrastructure of untreated *C. albicans* biofilms exhibited a dense cell network of yeasts, budding yeasts and hyphae. Treatment with 2, 4, 6-trimethoxybenzaldehyde significantly reduced cell density with few budded and hyphal forms (Figure 7).

**Figure 8:**
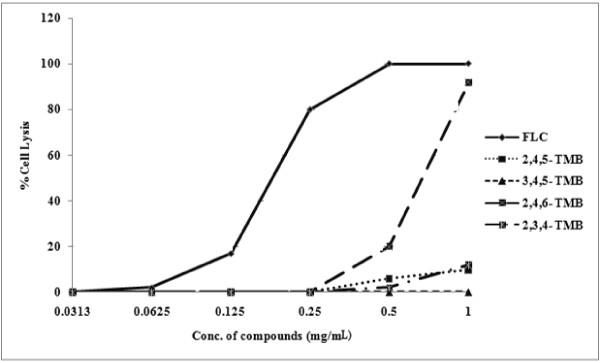
Hemolysis caused by 2,4,5-trimethoxybenzaldehyde; 2,3,4-trimethoxybenzaldehyde; 3,4,5-trimethoxybenzaldehyde; 2,4,6-trimethoxybenzaldehyde and fluconazole.

The *in vitro* hemolytic assay is a screening tool for gauging *in vivo* toxicity toward host cells [29]. The four compounds tested showed no significant toxicity to human erythrocytes at minimum growth inhibitory concentrations (*P* = 0.0303 for 2, 4, 5-trimethoxybenzaldehyde, *P* = 0.0331 for 2, 4, 5-trimethoxybenzaldehyde, *P* = 0.0396 for 2, 3, 4-trimethoxybenzaldehyde, and *P* = 0.040 for 3, 4, 5-trimethoxybenzaldehyde), whereas they had profound effects on dimorphism, adhesion and biofilm formation by *C. albicans* (Figure 8). Only 0–12% hemolysis was observed at the tested concentrations, while fluconazole showed 100% hemolysis at 0.5 mg/mL.

COX-dependent pathways may be responsible for the prostaglandin (PGE2) synthesis in *C. albicans*, and prostaglandin induces pathogenicity in *C. albicans*[30,31]. COX-II inhibitors inhibit morphogenesis and biofilm formation in *C. albicans*[32]. There is controversy about whether COX inhibitors affect filamentation by blocking prostaglandin synthesis and, moreover, it is known that *C. albicans* does not encode a COX homolog. Asaronaldehyde is a selective COX II inhibitor [33], and was found to inhibit yeast to hyphal transition and biofilm formation in this study.

**Figure 9:**
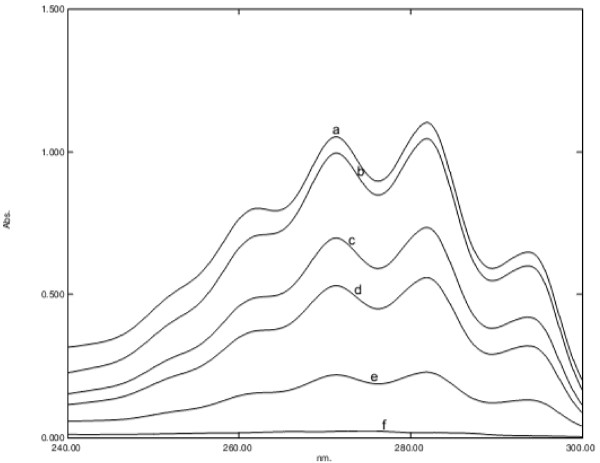
**UV spectrophotometric sterol profile of *C. albicans *ATCC 90028, treated with 0 (curve a), 0.0625 (curve b), 0.125 (curve c), 0.25 (curve d), 0.5 (curve e) and 1 (curve f) mg/mL of asaronaldehyde (2,4,5- trimethoxybenzaldehyde).**

In our study, asaronaldehyde inhibited sterol biosynthesis. As shown in Figure 9, the average decreases in total cellular ergosterol content of *Candida* cells after exposure to their respective MIC, MIC/2, MIC/4, MIC/8 and MIC/16 of 2, 4, 5-trimethoxybenzaldehyde were 92%, 47%, 25%, 10% and 0% respectively (*P* = 0.0453 for 2, 4, 5-trimethoxybenzaldehyde, *P* = 0.0406 for 2, 4, 5-trimethoxybenzaldehyde, *P* = 0.0397 for 2, 3, 4-trimethoxybenzaldehyde, and *P* = 0.0485 for 3, 4, 5-trimethoxybenzaldehyde). Other compounds did not show any effect on ergosterol biosynthesis. Additionally, beta-asarone, which is closely related to asaronaldehyde, inhibits ergosterol biosynthesis in *C. albicans*[34].

## Conclusion

Asaronaldehyde and 2, 4, 6-trimethoxybenzaldehyde showd anti-*Candida* efficacy.

## Abbreviations

COX: Cyclooxygenase.

## Competing interests

The authors declare that they have no competing interests.

## Authors’ contributions

KSM conceived the study. SBR and RS performed the experiments. SBR and MMR wrote the manuscript. All authors read and approved the final version of the manuscript.
